# Neonatal phototherapy and risk of epilepsy—A Danish population based study

**DOI:** 10.1007/s00431-024-05681-6

**Published:** 2024-07-06

**Authors:** Yuelian Sun, Julie Werenberg Dreier, Chunsen Wu, Jesper Padkær Petersen, Tine Brink Henriksen, Jakob Christensen, Rikke Damkjær Maimburg

**Affiliations:** 1 Department of Neurology, Department of Clinical Medicine, Aarhus University Hospital, Affiliated Member of the European Reference Network EpiCARE, Aarhus, Denmark; 2 Department of Clinical Epidemiology, Department of Clinical Medicine, Aarhus University Hospital, Aarhus University, Aarhus, Denmark; 3 National Centre for Register-Based Research, Department of Economics and Business Economics, Aarhus University, Aarhus, Denmark; 4 Centre for Integrated Register-Based Research (CIRRAU), Aarhus University, Aarhus, Denmark; 5 Department of Clinical Research, University of Southern Denmark, Odense, Denmark; 6 Department of Gynecology and Obstetrics, Odense University Hospital, Odense, Denmark; 7 Department of Pediatrics, Aarhus University Hospital, Aarhus, Denmark; 8 Department of Clinical Medicine, Aarhus University, Aarhus, Denmark; 9 Department of Clinical Medicine & Occupational Health, Aarhus University Hospital, Aarhus, Denmark; 10 Department of Midwifery, University College of Northern Denmark, Aalborg, Denmark; 11 School of Nursing and Midwifery. Western, Sydney University, Sydney, Australia

**Keywords:** Cohort, Epilepsy, Multivariable models, Neonatal hyperbilirubinemia, Neonatal phototherapy, Propensity score matching

## Abstract

To evaluate the risk of epilepsy in children who received neonatal phototherapy. A cohort of live singletons born at a Danish hospital (2002–2016) with a gestational age ≥ 35 weeks. We used Cox regression to estimate hazard ratios (HRs) and 95% confidence intervals (CIs) of epilepsy in children treated with neonatal phototherapy compared to children not treated with neonatal phototherapy in the general population, and in a subpopulation of children who had serum bilirubin measurement. Adjusted HRs (aHR) were computed using multivariable and propensity score matching models to take maternal and neonatal factors into consideration. Children were followed from day 29 after birth to diagnosis of epilepsy, death, emigration, or December 31, 2016. Among 65,365 children, 958 (1.5%) received neonatal phototherapy. Seven children (incidence rates (IRs): 10.8 /10,000 person-years) who received neonatal phototherapy and 354 children (IR: 7.7) who did not receive neonatal phototherapy were diagnosed with epilepsy. Neonatal phototherapy was not associated with an increased risk of epilepsy using the multivariable (aHR 0.95, 95% CI: 0.43–2.09) and propensity score matched (aHR 0.94, 95% CI: 0.39–2.28) models. In the subpopulation of 9,378 children with bilirubin measurement, 928 (9.9%) received neonatal phototherapy. In the analysis of the subpopulation in which bilirubin level and age at the time of bilirubin measurement were further taking into consideration, neonatal phototherapy was not associated with an increased risk of epilepsy using the multivariable (aHR 1.26, 95% CI: 0.54–2.97) and propensity score matched (aHR 1.24, 95% CI: 0.47–3.25) models,

*Conclusions*: Neonatal phototherapy was not associated with an increased risk of epilepsy after taking maternal and neonatal factors into consideration.

**Table:** 

**What is known:** • *A few studies have suggested that neonatal phototherapy for hyperbilirubinemia may increase the risk of childhood epilepsy.* • *Whether the observed associations contribute to hyperbilirubinemia, phototherapy, or underlying factors requires further investigation.*
**What is new:** • *This study revealed no increased risk of epilepsy in children treated with neonatal phototherapy compared to children not treated with phototherapy after taking maternal and neonatal factors into consideration.* • *After further taking bilirubin level and age at the time of bilirubin measurement into consideration, neonatal phototherapy was not associated with an increased risk of epilepsy.*

**Supplementary Information:**

The online version contains supplementary material available at 10.1007/s00431-024-05681-6.

## Introduction

Kernicterus, once prevalent due to neonatal hyperbilirubinemia, is now rare owing to improved care and treatment methods [1–5]. However, bilirubin neurotoxicity, encompassing a spectrum known as kernicterus spectrum disorder (KSD), remains a concern [1, 5, 6]. Phototherapy, alongside exchange transfusion in severe cases, is the standard treatment for hyperbilirubinemia in newborns [7, 8]. Despite its efficacy, concerns persist regarding potential adverse effects such as allergy, neurological disorders, and cancer [9–12]. Previous studies have suggested an increased risk of seizures or epilepsy in children undergoing neonatal phototherapy, yet determining causality between hyperbilirubinemia, neonatal phototherapy, and epilepsy remains challenging [10, 13]. In this study, we sought to contribute further evidence to this topic using Danish registry data from a university hospital.

## Methods

### Study design and study population

We conducted a cohort study utilizing data from the Danish Medical Birth Registry [14] to identify all live singleton births with a gestational age of ≥ 35 weeks at Aarhus University Hospital between January 1, 2002, and November 30, 2016. The study population comprised infants with available data on birth weight, who survived the neonatal period, and remained resident in Denmark on day 29 post-birth (*N* = 65,365; see Supplementary Fig. 1). In Denmark, each resident is assigned a unique identification number in the Danish Civil Registration System, facilitating accurate data linkage across national registers [15, 16]. Only children born at Aarhus University Hospital were included in this study due to incomplete information on neonatal phototherapy in other regions of Denmark [17]. We used data from the Danish registries and no consent forms are needed according to Danish data protection regulation. Our research adhered to the ethical principles outlined in the Declaration of Helsinki and the study was approved by the Danish Data Protection Agency (reference number: 2016–051-000001, serial number 1515, approval date: 11. June 2019). We utilized ChatGPT to assist in revising the language of our manuscript, but we carefully reviewed and incorporated the revisions.

### Information on measurements of bilirubin

Data on bilirubin measurements, including total serum bilirubin and unconjugated bilirubin, were extracted from the clinical laboratory information system (LABKA). Implemented in 2000 within the Central Denmark Region, LABKA offers comprehensive coverage within this region, encompassing Aarhus University Hospital [18]. The system aggregates test results from samples collected in both public and private hospitals, as well as those collected by general practitioners and submitted to clinical biochemistry departments.

Key test items during the neonatal period included total serum bilirubin (TsB) of neonates (NPU04145), total serum bilirubin (NPU01370), unconjugated bilirubin (NPU01366), and conjugated bilirubin (NPU17194), with the NPU terminology employed for result identification and communication across clinical laboratories [18].

The dataset contains details such as sampling date and time, results, and units of tests conducted. For this study, bilirubin measurement primarily pertains to total serum bilirubin and/or unconjugated bilirubin, given the negligible presence of conjugated bilirubin during the neonatal period. Multiple bilirubin measurements could be recorded for a child during this period, with the highest value selected for analysis.

### Information on neonatal phototherapy

Data on neonatal phototherapy treatment were extracted from the Danish National Patient Register (DNPR) [19]. Children receiving neonatal phototherapy were identified if they were coded with procedure codes BNGC (Phototherapy) or BNGC0 (Phototherapy to neonates) in the DNPR, with treatment dates falling within the neonatal period (up to 28 days of age). Clinical practices adhere to phototherapy guidelines for neonatal hyperbilirubinemia adapted by the Danish Pediatric Society in 1992 and 2012 from guidelines established by the American Academy of Pediatrics (see Supplementary Table 1a) [2, 20]. The information about the devices, light spectrum, intensity used for neonatal phototherapy at Aarhus University Hospital could be found in the Supplementary Table 1b. Since 2011, the Danish recommendation of intensity for neonatal phototherapy is a minimum 30 microwatts/cm^2^/nm, measured at initiation. Phototherapy usually last for 24 h before taking a bilirubin measurement to judge if the treatment should continue or not (personal communication with coauthor JPP).

### Information on diagnosis of epilepsy

Diagnoses of epilepsy were retrieved from the DNPR [21]. Children were classified as having epilepsy if they were registered with ICD-10 codes G40-G41 as either a primary or secondary diagnosis after the neonatal period. The date of epilepsy onset was defined as the date of hospital contact that led to the diagnosis.

### Information on covariates

Data on date and time of birth, sex of the child, gestational age at birth, birth weight, Apgar score at five minutes, maternal age at the time of birth, and parity were obtained from the Danish Medical Birth Registry. The age of the child in hours at the time of blood sample collection for bilirubin tests was determined by utilizing both the date and time of birth and blood sample collection.

Children were classified based on gestational age and birth weight percentile: those with a birth weight below the 10th percentile for their gestational age were categorized as small for gestational age (SGA), those with a birth weight above the 10th percentile were classified as large for gestational age (LGA), and those with a birth weight between the 10th and 90th percentile were deemed appropriate for gestational age (AGA). Information on family disposable income was obtained from Statistics Denmark [22].

Diagnoses of major congenital malformations in the neonatal period (within 28 days after birth) were extracted from the DNPR. Information on major congenital malformations (see Supplementary Table 2), other neonatal factors (see Supplementary Table 3) during the neonatal period, as well as maternal factors during pregnancy (see Supplementary Table 4), were also obtained from the DNPR.

### Statistics

Characteristics of children were summarized for children with neonatal phototherapy and children without neonatal phototherapy among the total population and among children with available measurement of bilirubin. For children with measurement of bilirubin, we estimated mean and standard deviation of the maximum of bilirubin measured in the neonatal period and the age of neonates at time of bilirubin measurement.

Crude and adjusted hazard ratios (HRs) of epilepsy for children with neonatal phototherapy compared to those without neonatal phototherapy were estimated using Cox proportional regression models. Children were followed from day 29 after birth until the onset of epilepsy, death, emigration, or the end of follow-up on December 31, 2016. Age served as the time scale in the Cox proportional regression models.

Adjusted HRs of epilepsy were calculated in two ways, the traditional multivariable model and propensity score matching model, since the propensity score matching model enable us to take more covariates into account than the traditional one. In the analyses using the multivariable model [23], each factor (including sex, gestational age, intrauterine growth [SGA, AGA, LGA], congenital malformation, Apgar score, neonatal and maternal risk factors, birth year, family income, maternal age, and parity) was included as an independent variable. In the analyses using propensity score matching, propensity scores of neonatal phototherapy were computed based on child and maternal risk factors (27 in total, see Table 1). We employed a 1:3 ratio using nearest neighbor matching with a caliper of 0.1 of the standard deviation of the logit of the propensity score and ensured common support of propensity score (overlap in the propensity score distribution between the treatment and control groups) [24]. We did balancing test of neonatal and maternal factors between children with neonatal phototherapy and children without neonatal phototherapy before and after propensity score matching.

**Table 1:** Characteristics of children according to neonatal phototherapy among the total population (*n* = 65,365) and among children with available measurement of bilirubin (*n* = 9,378)

Characteristics	Total population (*n* = 65,365)				Sub-population of children with measurement of bilirubin (*n* = 9,378)			
	Children without neonatal phototherapy (*n* = 64,407, 98.5%)		Children with neonatal phototherapy (*n* = 958, 1.5%)		Children without neonatal phototherapy (*n* = 8,450, 90.1%)		Children with neonatal neonatal phototherapy ^e^ (*n* = 928, 9.9%)	
	No	%	No	%	No	%	No	%
Sex ^a^								
Girls	31,544	49.0	403	42.1	3,690	43.7	391	42.1
Boys	32,863	51.0	555	57.9	4,760	56.3	537	57.9
Gestational age (weeks) ^a^								
35	537	0.8	216	22.6	393	4.7	216	22.6
36	1,224	1.9	185	19.3	665	7.9	185	19.3
37	3,198	5.0	166	17.3	1,066	12.6	166	17.3
38	8,877	13.8	155	16.2	1,755	20.8	155	16.2
> = 39	50,571	78.5	236	24.6	4,571	54.1	236	24.6
Growth of neonates ^a^								
AGA ^b^	52,443	81.4	672	70.2	6,585	77.9	672	70.2
SGA ^b^	5,764	9.0	157	16.4	907	10.7	157	16.4
LGA ^b^	6,200	9.6	129	13.5	958	11.3	129	13.5
Major congenital malformation in neonatal period ^a^								
No	63,111	98.0	888	92.7	7,983	94.5	863	93.0
Yes	1,296	2.0	70	7.3	467	5.5	65	7.0
Apgar score at 5 min ^a^								
09–10	62,157	96.5	872	91.0	7,814	92.5	872	91.0
7–8	1,299	2.0	51	5.3	379	4.5	51	5.3
0–6	222	0.3	13	1.4	105	1.2	13	1.4
Missing	729	1.1	22	2.3	152	1.8	22	2.3
Any other factors of child in the neonatal period								
No	58,333	90.6	566	59.1	6,253	74.0	552	59.5
Yes	6,074	9.4	392	40.9	2,197	26.0	376	40.5
Birth asphyxia ^a^								
No	63,281	98.3	910	95.0	8,000	94.7	910	95.0
Yes	1,126	1.8	48	5.0	450	5.3	48	5.0
Acidosis ^a^								
No	63,863	99.2	945	98.6	8,250	97.6	915	98.6
Yes	544	0.8	13	1.4	200	2.4	13	1.4
Infection ^a^								
No	62,729	97.4	879	91.8	7,769	91.9	879	91.8
Yes	1,678	2.6	79	8.3	681	8.1	79	8.3
Birth injury ^a^								
No	64,149	99.6	943	98.4	8,370	99.1	913	98.4
Yes	258	0.4	15	1.6	80	1.0	15	1.6
Syndrome of infants of mother with diabetes ^a^								
No	63,416	98.5	865	90.3	8,068	95.5	840	90.5
Yes	991	1.5	93	9.7	382	4.5	88	9.5
Hypoglycaemia ^a^								
No	63,061	97.9	808	84.3	7,917	93.7	787	84.8
Yes	1,346	2.1	150	15.7	533	6.3	141	15.2
Underfeeding ^a^								
No	64,120	99.6	927	96.8	8,282	98.0	897	96.7
Yes	287	0.5	31	3.2	168	2.0	31	3.3
Respiratory disorder ^a^								
No	61,842	96.0	800	83.5	7,563	89.5	800	83.5
Yes	2,565	4.0	158	16.5	887	10.5	158	16.5
Cardiovascular disorder ^a^								
No	64,248	99.8	943	98.4	8,357	98.9	913	98.4
Yes	159	0.3	15	1.6	93	1.1	15	1.6
Neonatal convulsion or intracranial_haemorrhage ^a^								
No	64,299	99.8	952	99.4	8,369	99.0	922	99.4
Yes	108	0.2	6	0.6	81	1.0	6	0.7
Any of maternal risk factors during pregnancy								
No	47,663	74.0	479	50.0	5,423	64.2	464	50.0
Yes	16,744	26.0	479	50.0	3,027	35.8	464	50.0
Preeclampsia ^a^								
No	61,824	96.0	850	88.7	7,889	93.4	850	88.7
Yes	2,583	4.0	108	11.3	561	6.6	108	11.3
Diabetes ^a^								
No	61,726	95.8	835	87.2	7,761	91.9	811	87.4
Yes	2,681	4.2	123	12.8	689	8.2	117	12.6
Hemorrhage in early pregnancy ^a^								
No	61,999	96.3	912	95.2	8,110	96.0	912	95.2
Yes	2,408	3.7	46	4.8	340	4.0	46	4.8
Antepartum/Intrapartum hemorrhage ^a^								
No	63,223	98.2	921	96.1	8,238	97.5	921	96.1
Yes	1,184	1.8	37	3.9	212	2.5	37	3.9
Infection of genitourinary tract in pregnancy ^a^								
No	61,081	94.8	880	91.9	7,942	94.0	880	91.9
Yes	3,326	5.2	78	8.1	508	6.0	78	8.1
Premature rupture of membrane ^a^								
No	58,252	90.4	768	80.2	7,290	86.3	768	80.2
Yes	6,155	9.6	190	19.8	1,160	13.7	190	19.8
Infection of amniotic sac and membrane ^a^								
No	63,819	99.1	943	98.4	8,350	98.8	943	98.4
Yes	588	0.9	15	1.6	100	1.2	15	1.6
Placenta praevia or abruption placentae ^a^								
No	63,903	99.2	939	98.0	8,345	98.8	909	98.0
Yes	504	0.8	19	2.0	105	1.2	19	2.1
Birth year ^a^								
2002–2006	21,182	32.9	288	30.1	2,331	27.6	277	29.9
2007–2011	21,979	34.1	332	34.7	2,693	31.9	318	34.3
2012–2016	21,246	33.0	338	35.3	3,426	40.5	333	35.9
Family income (quartiles) ^a^								
1	17,472	27.1	271	28.3	2,292	27.1	271	28.3
2	13,654	21.2	245	25.6	1,880	22.3	245	25.6
3	15,206	23.6	236	24.6	1,971	23.3	236	24.6
4	18,075	28.1	206	21.5	2,307	27.3	206	21.5
Maternal age (years) ^a^								
< 25	6,323	9.8	97	10.1	881	10.4	97	10.1
25–29	21,084	32.7	333	34.8	2,868	33.9	333	34.8
30–34	24,305	37.7	343	35.8	2,961	35.0	343	35.8
35–39	10,616	16.5	146	15.2	1,432	17.0	146	15.2
> = 40	2,079	3.2	39	4.1	308	3.7	39	4.1
Parity ^a^								
Nulliparous ^c^	31,531	49.0	536	56.0	4,797	56.8	519	55.9
Multiparous	32,876	51.0	422	44.1	3,653	43.2	409	44.1
Age at time of bilirubin measurement ^d^								
1st day					265	3.1	18	1.9
2nd day					731	8.7	66	7.1
3rd day					2,294	27.2	202	21.8
4th day					1,359	16.1	243	26.2
5–7 days					1,674	19.8	276	29.7
8–14 days					909	10.8	105	11.3
15–21 days					790	9.4	12	1.3
22–28 days					428	5.1	6	0.7
Gestational age- and age-specific quartile of bilirubin ^d^								
1st					2,260	26.8	49	5.3
2nd					2,229	26.4	110	11.9
3rd					2,146	25.4	197	21.2
4th					1,815	21.5	572	61.6

^a^^:^ factors included in calculating propensity score in the analyses among the total population

^b^^:^ AGA: appropriate for gestational age; SGA: small for gestational age; LGA: large for gestational age

^c^^:^ children with a missing value of parity (n = 248) was categorized into the group of children born to nulliparous mothers

^d^^:^ extra factors included in calculating propensity score in the analyses among the subpopulation of children with bilirubin measurement besides the factors marked with ^a^ in Table 1

^e^^:^ For the factors (including gestational age, growth of neonates, apgar score at 5 min, birth asphyxia, infection, respiratory disorder, preeclampsia, hemorrhage in early pregnancy, antepartum / intrapartum hemorrhage, infection of genitourinary tract in pregnancy, premature rupture of membrane, family income, maternal age), we presented information of children with neonatal phototherapy from the total population to replace the information of children with neonatal phototherapy from subpopulation, who had bilirubin measurement, due to data protection regulations, as differences in some categories between the total population and the sub-population were < 5. The true numbers were used in the analyses

We conducted similar analyses in the subgroup of children who had at least one bilirubin measurement in the neonatal period enabling us to further adjust for bilirubin related factors and estimate the influence of phototherapy itself on epilepsy risk. In the multivariable model, we further adjusted for gestational age- and age-specific quartiles of bilirubin level (1st, 2nd, 3rd, 4th quartile) and age at the time of bilirubin measurement (1st, 2nd,3rd, 4th, 5–7, 8–14, 15–21, and 22–28 days after birth). For propensity score matching, we included gestational age- and age-specific quartiles of bilirubin level and age at the time of bilirubin measurement in the calculation of the propensity score of neonatal phototherapy (see Table 1). To obtain gestational age- and age-specific quartiles of bilirubin, children were categorized into quartiles within each stratum based on gestational age (35, 36, 37, 38, or ≥ 39 gestational weeks) and age at the time of bilirubin measurement (< 48 h, 48–71 h, 72–95 h, 4–14 days, and 15–28 days).

All analyses were performed with STATA, version 16 (StataCorp LLC, College Station, TX, USA).

## Results

**Fig. 1:**
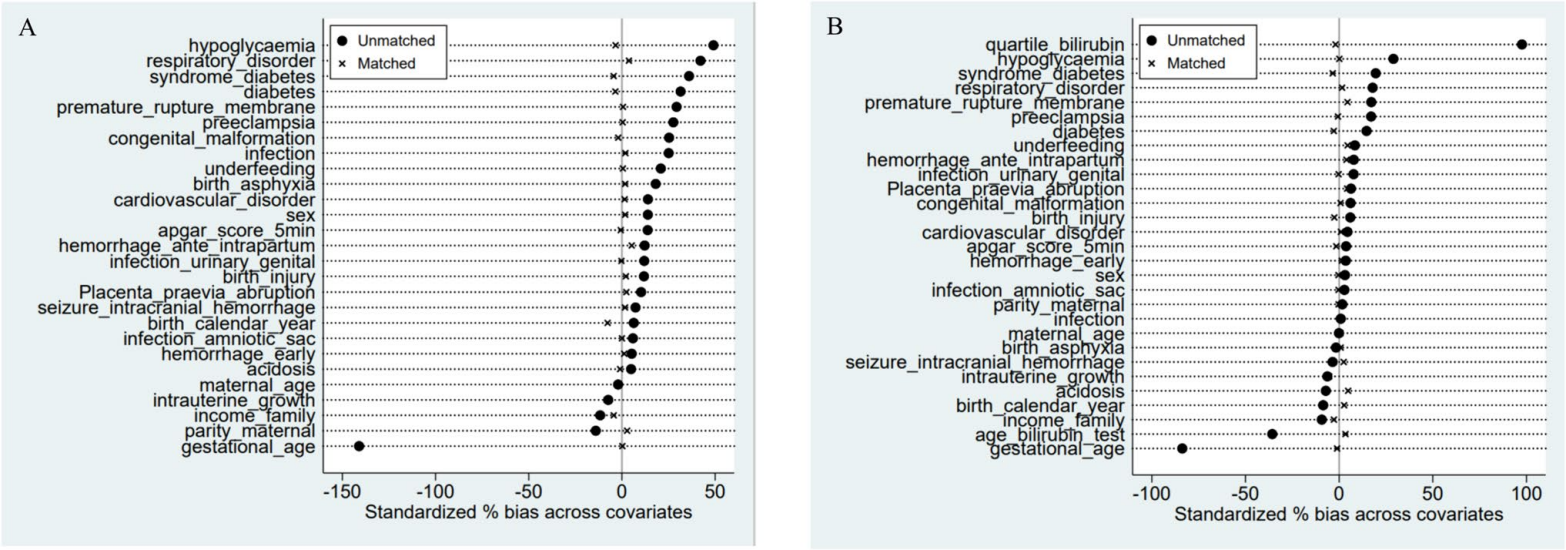
Balancing test (standardized % bias) of child and maternal factors between children with neonatal phototherapy and children without neonatal phototherapy before (marked with a solid black dot) and after (marked with a cross) propensity score matching in the total population (A) and in a sub-population of children with a measurement of bilirubin in the neonatal period (B). In the analyses for the sub-population, two more factors were adjusted for including bilirubin level (quartile_bilirubin) and neonatal age at time of bilirubin measurement (age_bilirubin_test). The label of each factor and balancing test of these factors before and after propensity score matching in detail is presented at Supplementary Table 5

In the total study population (*n* = 65,365), 958 (1.5%) children received neonatal phototherapy treatment in the neonatal period. Children undergoing neonatal phototherapy exhibited a higher prevalence of adverse conditions compared to those without neonatal phototherapy (Table 1). Over a mean follow-up period of 7.2 years (up to 15 years), seven children (incidence rate [IR] 10.8/10,000 person-years) were diagnosed with epilepsy among those receiving neonatal phototherapy, while 354 children (IR 7.7/10,000 person-years) were diagnosed with epilepsy among those without neonatal phototherapy. The adjusted hazard ratio (HR) for epilepsy in children with neonatal phototherapy compared to those without was 0.95 (95% CI: 0.43–2.09) in the multivariable model and 0.94 (95% CI: 0.39–2.28) in the propensity matching model (Table 2). In the analyses using the propensity score matching model, child and maternal factors were balanced between children with and without neonatal phototherapy (Fig. 1A, Supplementary Table 5).

**Table 2:** Hazard ratio of epilepsy for children with neonatal phototherapy compared with children without neonatal phototherapy (n = 65,365) in the Cox regression analyses with multivariable model and propensity score matching model for confounders adjustment

Neonatal phototherapy	No	Person years	Epilepsy	IR / 10,000 pys ^a^	Crude HR	Adjusted HR
Multivariable model						
No	64,407	458,221	354	7.7	1.00 (ref)	1.00 (ref)
Yes	958	6,503	7	10.8	1.38 (0.65—2.92)	0.95 (0.43—2.09) ^b^
Propensity score ^c^ matching model						
No	2,197	14,731	17	11.5	-	1.00 (ref)
Yes	958	6,503	7	10.8	-	0.94 (0.39–2.28)

^a^^:^ IR: incidence rate; pys: person years

^b^^:^ adjusted for sex, gestational age (35,36,37,38, ≥ 39 weeks), intrauterine growth (SGA, AGA, LGA), congenital malformation diagnosed in the neonatal period (yes, no), Apgar score at 5 min (9–10, 7–8, 0–6, missing), other child risk factors in the neonatal period (yes, no), maternal risk factors (yes, no), birth year (2002–2006, 2007–2011, 2012–2016), family income (quartiles), maternal age (< 25, 25–29, 30–34,35–39, ≥ 40), parity (Nulliparous, multiparous)

^c^^:^ Factors in calculation of propensity score of neonatal phototherapy are listed in Table 1, which are marked with ^a^

In the subpopulation (*n* = 9,378) with bilirubin measurement in the neonatal period, 928 (9.9%) children received neonatal phototherapy. Similarly, children with neonatal phototherapy exhibited a higher prevalence of adverse conditions compared to those without neonatal phototherapy, except for birth asphyxia, acidosis, and neonatal convulsion/intracranial hemorrhage, where children with neonatal phototherapy showed lower prevalence rates of these factors (Table 1). Children who underwent neonatal phototherapy had higher bilirubin levels compared to children without neonatal phototherapy (Table 3). Bilirubin measurements for children with neonatal phototherapy were more likely to occur around 4–7 days after birth, while children without neonatal phototherapy had a more varied distribution of bilirubin measurements (Table 1, Table 3). During follow-up, seven children (IR 11.2/10,000 person-years) were diagnosed with epilepsy among those receiving neonatal phototherapy, compared to 86 children (IR 15.9/10,000 person-years) among those without neonatal phototherapy. The adjusted HR for epilepsy in children with neonatal phototherapy compared to children without neonatal phototherapy was 1.26 (95% CI: 0.54–2.97) in the multivariable model and 1.24 (95% CI: 0.47–3.25) in the propensity matching model (Table 4). In the analyses using the propensity score matching model, child and maternal factors were balanced between children with and without neonatal phototherapy (Fig. 1B, Supplementary Table 5).

**Table 3:** The distribution (mean and standard deviation, SD) of the maximum of bilirubin and the age of neonates when bilirubin was measured according to gestational age and neonatal phototherapy

Gestational age (weeks)	Number of children	Children with measurement of bilirubin		Maximum of bilirubin in the neonatal period (umol/l)		Age (days) at time of bilirubin test	
	No	No	(row%)	Mean	(SD)	Mean	(SD)
Children without neonatal phototherapy							
Total	64,407	8,450	(13.1)				
35–36	1,761	1,058	(60.1)	225	(66.8)	4.9	(4.0)
37–38	12,075	2,821	(23.4)	219	(76.8)	6.7	(6.2)
39–45	50,571	4,571	(9.0)	190	(80.8)	6.1	(6.5)
Children with neonatal phototherapy							
Total	958	928	(96.7)				
35–36	401	394	(98.3)	277	(53.1)	4.7	(3.0)
37–38	321	310	(96.6)	315	(78.2)	4.3	(2.6)
39–45	236	224	(94.9)	315	(81.5)	3.8	(2.9)

**Table 4:** Hazard ratio of epilepsy among children treated with neonatal phototherapy compared to children without neonatal phototherapy among children with a measurement of bilirubin in the neonatal period (n = 9,378) in the Cox regression analyses with multivariable model and propensity score matching model for confounders adjustment

Neonatal phototherapy	No	Person years	Epilepsy	IR / 10,000 pys ^a^	Crude HR	Adjusted HR
Multivariable model						
No	8,450	53,979	86	15.9	1.00 (ref)	1.00 (ref)
Yes	928	6,258	7	11.2	0.72 (0.33—1.56)	1.26 (0.54—2.97) ^b^
Propensity score matching model ^c^						
No	1,616	10,687	10	9.4	-	1.00 (ref)
Yes	919	6,184	7	11.3	-	1.24 (0.47—3.25)

^a^^:^ IR: incidence rate ratio; pys: person years

^b^^:^ adjusted for sex, gestational age (35,36,37,38, ≥ 39 weeks), intrauterine growth (SGA, AGA, LGA), congenital malformation diagnosed in the neonatal period (yes, no), Apgar score (9–10, 7–8, 0–6, missing), other child risk factors in the neonatal period (yes, no), maternal risk factors (yes, no), birth year (2002–2006, 2007–2011, 2012–2016), family income (quartiles), maternal age (< 25, 25–29, 30–34,35–39, ≥ 40), parity (Nulliparous, multiparous), age of child at time of bilirubin measurement, and gestational age- and age-specific quartile of bilirubin

^c^^:^ Factors in calculation of propensity score of neonatal phototherapy are listed in Table 1, which are marked with ^a or d^

## Discussion

This study showed that children who underwent neonatal phototherapy did not have an increased risk of epilepsy compared to children without neonatal phototherapy in the general population. We also found no increased risk of epilepsy associated with neonatal phototherapy in children who had bilirubin measurements, in which bilirubin level and age at the time of bilirubin measurement were further taken into consideration besides many other maternal and neonate’s factors. The finding was robust when accounting for potential confounders using different statistical models.

Our study findings diverged from those of previous studies conducted in Denmark [13] and the US [10]. Maimburg et al. (2016) reported a 1.6-fold (95%CI: 1.23–2.24) increased risk of epilepsy among children who underwent neonatal phototherapy, particularly in boys [13], whereas Newman et al. (2018) found a 1.3-fold (95%CI: 1.10–1.61) increased risk of seizures in boys who received neonatal phototherapy compared to those who had bilirubin measurements but did not receive neonatal phototherapy [10]. Methodological differences and variations in healthcare systems may contribute to these discrepancies. The study by Maimburg et al. (2016) collected information on neonatal phototherapy reported by mothers and included children born both ≥ 35 gestational weeks and below 35 gestational weeks [13]. The primary outcome in the US study by Newman et al. (2018) was defined as those with at least one seizure diagnosis and at least one antiseizure medication prescription, providing a slightly different perspective [10]. Notably, the proportion of children receiving neonatal phototherapy in the US study (7.6%) was higher than in ours (1.5% in the study population in which neonatal death were not included and 1.6% in the general population [17]), despite both focusing on children born ≥ 35 gestational weeks. Furthermore, the proportion of children receiving neonatal phototherapy in the US study increased significantly from 2.4% to 15.9% during the study period (1995–2011), [25] whereas the proportion in our study remained stable during the study period (2002–2016) [17]. Fortunately, we could conduct similar analyses by restricting to children with bilirubin measurement, akin to the US study.

The lower epilepsy incidence rate (unadjusted) and a non-significant increased risk after adjusting for potential confounders among children with bilirubin measurements undergoing neonatal phototherapy compared to children with bilirubin measurements without neonatal phototherapy, present an intriguing observation. It emphasizes the importance of adjusting for confounders and considering the complex interplay between phototherapy, bilirubin levels, and other potential risk factors for neurological outcomes. While phototherapy effectively reduces bilirubin levels, thereby mitigating the risk of bilirubin-induced neurotoxicity, it's possible that other mechanisms or confounding variables influence the risk of epilepsy independently [26].

Our study possessed both strengths and limitations. It was a population-based study with nearly complete follow up. Both information on neonatal phototherapy and serum bilirubin levels were derived from registers and recall bias was unlikely. The register provided detailed information on bilirubin measurements, enabling adjustment for bilirubin levels and measurement timing. However, information on the timing, duration, intensity, and frequency of neonatal phototherapy in our study was lacking, which may have impacted our ability to assess its effects comprehensively, for example dose–response relationship between neonatal phototherapy and risk of epilepsy. A Japanese observational cohort study demonstrated that a long duration of neonatal phototherapy was positively associated with the risk of allergic disorders in childhood [27]. Our study benefited from rich data on pregnancy, birth, and the post-natal period for both mothers and children. We employed two methods to adjust for potential confounders, including multivariable regression modeling and propensity score matching, with consistent findings. Our study's small sample size resulted in limited statistical power and our ability to conduct additional analyses, like stratification analyses for boys and girls. Perhaps therefore, this study was not able to assess previous findings of an increased risk of epilepsy among boys treated with neonatal phototherapy. [10, 13] While previous research has shown inconclusive results regarding a link between neonatal phototherapy and childhood neurological development, including epilepsy, our study adds to the growing body of evidence suggesting minimal to no risk of adverse neurocognitive effects. [28] However, larger and more comprehensive data sets, including timing and dose in terms of duration, intensity, and frequency of neonatal phototherapy are needed to elucidate relationship between neonatal phototherapy and children neurological development. Additionally, applying methodologies such as regression discontinuity design [29, 30] could provide rigorous causal inference regarding the safety of neonatal phototherapy on child neurological development [31–34]. Notably, our study took place in a setting where neonatal phototherapy was maintained at a reasonable low level (1.6% children born at 35 gestational weeks and later were registered with neonatal phototherapy). [17] Avoiding overtreatment of neonatal phototherapy may be recommended both to reduce health care costs and potential adverse effects, without increasing the rates of kernicterus. [28, 35]

## Conclusions

Our study, based on a population-based cohort in Denmark, neonatal phototherapy for neonatal hyperbilirubinemia was not associated with an increased risk of epilepsy. The study had limited power, and further research is warranted to clarify the relationship between neonatal phototherapy and epilepsy risk.

### Supplementary Information

Below is the link to the electronic supplementary material.

Supplementary file1 (DOCX 80.5 KB)

## Data Availability

The original data cannot be shared for data protection reasons. For questions on the original data, please contact Yuelian Sun, email: ys@clin.au.dk.

## Abbreviations

AGA: Appropriate for gestational age
CI: Confidence interval
DNPR: Danish National Patient Register
HR: Hazard ratio
ICD: International Classification of Diseases
LABKA: Clinical laboratory information system
LGA: Large for gestational age
SGA: Small for gestational age
TsB: Total serum bilirubin
