# Supplementary material for: Neonatal phototherapy and risk of epilepsy—A Danish population based study
Source: Eur J Pediatr. 2024 Jul 6;183(9):4111–21. doi: 10.1007/s00431-024-05681-6 (PMC11322204; doi:10.1007/s00431-024-05681-6)
Supplement: Supplementary file 1 — Supplementary file1 (DOCX 80.5 KB) [file 431_2024_5681_MOESM1_ESM.docx]

Euro J Pediatrics

Supplementary Information

**Neonatal Phototherapy and Risk of Epilepsy**

**- a Danish Population Based Study**

Yuelian Sun^1,2,3^, PhD, Julie Werenberg Dreier^3,4^, PhD, PhD, Chunsen Wu, PhD^5,6^, PhD, Jesper Padkær Petersen^7^, PhD, Tine Brink Henriksen^7,8^, PhD, Jakob Christensen, PhD^1,3,8*^, PhD, Rikke Damkjær Maimburg^9,10,11*^, PhD

^1^ Department of Neurology, Department of Clinical Medicine, Aarhus University Hospital, Affiliated Member of the European Reference Network EpiCARE, Aarhus, Denmark

^2^ Department of Clinical Epidemiology, Department of Clinical Medicine, Aarhus University Hospital, Aarhus University, Aarhus, Denmark

^3^ National Centre for Register-Based Research, Department of Economics and Business Economics, Aarhus University, Aarhus, Denmark

^4^ Centre for Integrated Register-Based Research (CIRRAU), Aarhus University, Aarhus, Denmark

^5^ Department of Clinical Research, University of Southern Denmark, Odense, Denmark

^6^ Department of Gynecology and Obstetrics, Odense University Hospital, Odense, Denmark

^7^ Department of Pediatrics, Aarhus University Hospital, Aarhus, Denmark

^8^ Department of Clinical Medicine, Aarhus University, Aarhus, Denmark

^9^ Department of Clinical Medicine & Occupational Health, Aarhus University hospital, Aarhus, Denmark

^10^ Department of Midwifery, University College of Northern Denmark, Aalborg, Denmark

^11^ School of Nursing and Midwifery. Western Sydney University, Sydney, Australia

**Address correspondence to:** Yuelian Sun, Olof Palmes Allé 43-45, Aarhus N 8200, Denmark, Email: [ys@clin.au.dk](mailto:ys@clin.au.dk)

Supplementary Table 1a. Information on guidelines of phototherapy treatment for neonatal hyperbilirubinemia developed by the Danish Pediatric Society in 1992 and 2012

| Guidelines issued in 1992 | Guidelines issued in 2012 |
| --- | --- |
| Threshold of bilirubin for phototherapy corresponds to 10% of the birthweight in grams, with a maximum of 300 umol/L for mildly affected newborns.^2^  For example if a child had a birth weight of 2800 gram, children who have a measurement of bilirubin of 280 umol/L or above should receive phototherapy. | Threshold of bilirubin for phototherapy is based on the age of the child when bilirubin was measured and birth weight.  The bilirubin levels used to define the need for phototherapy is 350, 250, 200, and 100 umol/L for children with a birth weight of >2500, 1500-2499, 1000-1499, or <1000 g if the bilirubin measurement was taken at age of 72 hours or older and 300 umol/L for children born at gestational age 34-36 weeks and a birth weight of >2500 g. ^18^ |

350 umol/L=20.5 mg/dL

300 umol/L=17.5 mg/dL

250 umol/L=14.6 mg/dL

200 umol/L=11.7 mg/dL

100 umol/L=5.8 mg/dL

Supplementary Table 1b. Main devices^a^ used for neonatal phototherapy at Aarhus University Hospital during the study period

| Devices | Manufacturer | Operate spectrum (nm) | Intensity ^d^ (microwatts/cm²/nm) | Filtered light |
| --- | --- | --- | --- | --- |
| Between 2002-2010/11, the following devices^b^ were used at the hospital | | | |  |
| Photo therapy 800 | Dräger, Germany | unknown | not measured regularly | yes |
| Photo-Therapy 4000 | Dräger, Germany | 450-475 | not measured regularly | yes |
|  |  |  |  |  |
| From 2010/11 onwards, the following devices ^c^ were used at the hospital | | | |  |
| Fiber Optic Pad Large Bilisoft model M1093119 | GE Healthcare | 450-475 | 40-48 | yes |
| BiliCocoon Light Box | NeoMedLight | 450-475 | 40-48 | yes |
| Neoblue mini LED | Natus Medical | 450-475 | 35 | yes |
| Neoblue LED | Natus Medical | 450-475 |  | yes |
| ^a^ It is estimated by the author (JPP) that the devices listed cover more than 98% of treatments. | | | | |
| ^b^ The second device replaced the first one during 2002-2010/2011, but it is not clear when it occurred. | | | | |
| ^c^ These devices were introduced at different times in the hospital and might have overlapping usage periods. | | | | |
| ^d^ Since 2011, the Danish recommendation has been for phototherapy to be delivered with a minimum intensity of 30 microwatts/cm²/nm, measured at initiation. | | | | |

Supplementary Table 2: ICD-10 codes for minor congenital malformations that were excluded when we identified children with a major congenital malformation (ICD-10: Q00-Q99)

| Location | ICD-10 codes |
| --- | --- |
| Head | Q671 Q672 Q189 Q670 Q673 Q753 Q674 |
| Eyes | Q135 Q101 Q102 Q752 Q103 Q105 Q078B Q188A |
| Ears | Q170 Q173 Q175 Q170 Q173 Q174 Q171 Q172 Q173 Q181 Q173 Q179 |
| Nose | Q6741 Q189 |
| Oral regions | Q3850 Q186 Q382 Q184 Q187 Q185 Q674 Q381 |
| Neck | Q189 Q182 Q181 Q180 Q680 |
| Hands | Q7400 Q6810 Q8280 Q845 |
| Feet limb | Q653 Q654 Q655 Q656 Q668 Q669 Q665 Q845 Q663 Q666 Q662 Q667 Q664 |
| Skin | Q833 Q825 Q8252 Q8250 Q8251 |
| Skeletal | Q7660 Q7662 Q7643 Q6821 Q7671 Q765 Q683 Q684 Q685 Q675 Q676 Q677 Q678 Q760 |
| Brain | Q0461 |
| Cardiovascular | Q270 Q261 Q2111 Q2541 Q250 Q256 |
| Pulmonary | Q331 Q314 Q315 Q320 Q3310 |
| Gastro-intestinal | Q4021 Q4320 Q4381 Q4382 Q401 Q430 Q400 |
| Renal | Q633 Q610 Q627 |
| External genitals | Q5521 Q5520 Q527 Q525 Q523 Q53 |
| Other | Q899 Q950 Q951 |

Supplementary Table 3: ICD 10 codes for diseases or status in the neonatal period

| Diseases or status in the neonatal period | ICD 10 codes |
| --- | --- |
| Acidosis | E872 |
| Birth asphyxia | P21 |
| Infection | P35-P39 |
| Birth injury | P10-P15 |
| Syndrome of infant of mother with diabetes | P70.0-P70.1 |
| Neonatal hypoglycemia | P70.4 |
| Underfeeding | P92.3 |
| Neonatal convulsion | P90 |
| Respiratory disorders | P22-P28 |
| Cardiovascular disorders | P29 |
| Intracranial nontraumatic hemorrhage | P52 |

We identified children with the risk factors if they had a primary or secondary diagnosis of the disorder in the neonatal period in the DNPR

Supplementary Table 4: ICD 10 codes for maternal disorders during pregnancy

| Diseases or status in the neonatal period | ICD 10 codes |
| --- | --- |
| Preeclampsia | O14 |
| Diabetes in pregnancy | O24 |
| Daemorrhage in early pregnancy | O20 |
| Antepartum haemorrhage | O46 |
| Infection of genitourinary tract | O23 |
| Premature rupture of membrane | O42 |
| Intrapartum haemorrhage | O67 |
| Infection of amniotic sac and membranes | O41 |
| Placenta praevia | O44 |
| Abruption placentae | O45 |

We identified children exposed to maternal risk factors during pregnancy if mother had a primary or secondary diagnosis of the disorder.

Supplementary Table 5. Balancing test of child and maternal factors between children with phototherapy and children without phototherapy before and after propensity score matching (U for unmatched, M for matched)

| Variable |  | Total population | |  |  |  | |  |  | Sub-population of children with measurement of bilirubin | | | | | |
| --- | --- | --- | --- | --- | --- | --- | --- | --- | --- | --- | --- | --- | --- | --- | --- |
| (Label of variable) |  | Mean ^a^ |  | %bias ^b^ | %reduct | t-test | |  |  | Mean |  | %bias | %reduct | t-test |  |
|  |  | Children with phototherapy | Children without phototherapy |  | \|bias\| | | t | p>\|t\| |  | Children with phototherapy | Children without phototherapy |  | \|bias\| | t | p>\|t\| |
| sex | U | 1,5793 | 1,5102 | 13.9 |  | 4.25 | | 0.000 |  | 1,5787 | 1,5633 | 3.1 |  | 0.90 | 0.371 |
| (Sex) | M | 1,5793 | 1,5706 | 1.8 | 87.4 | 0.38 | | 0.700 |  | 1,5789 | 1,5807 | -0.4 | 88.2 | -0.08 | 0.937 |
|  |  |  |  |  |  |  | |  |  |  |  |  |  |  |  |
| gestational_age | U | 37.01 | 38.673 | -141.0 |  | -68.32 | | 0.000 |  | 36.99 | 38.118 | -83.8 |  | -26.88 | 0.000 |
| (Gestationa age) | M | 37.01 | 37.008 | 0.2 | 99.8 | 0.04 | | 0.967 |  | 37.008 | 37.022 | -1.1 | 98.7 | -0.22 | 0.829 |
|  |  |  |  |  |  |  | |  |  |  |  |  |  |  |  |
| intrauterine_growth | U | 1,9708 | 2,0068 | -7.3 |  | -2.56 | | 0.011 |  | 1,974 | 2.006 | -6.3 |  | -1.93 | 0.053 |
| (Intrauterine growth) | M | 1,9708 | 2,0104 | -8.1 | -10.2 | -1.67 | | 0.096 |  | 1.975 | 2,002 | -5.4 | 14.7 | -1.13 | 0.257 |
|  |  |  |  |  |  |  | |  |  |  |  |  |  |  |  |
| congenital_malformation | U | .07307 | .02012 | 25.3 |  | 11.38 | | 0.000 |  | .07004 | .05527 | 6.1 |  | 1.85 | 0.065 |
| (Congenital_malformation) | M | .07307 | .07724 | -2.0 | 92.1 | -0.35 | | 0.729 |  | .06855 | .06674 | 0.7 | 87.7 | 0.15 | 0.877 |
|  |  |  |  |  |  |  | |  |  |  |  |  |  |  |  |
| apgar_score_5min | U | 1,264 | 1,118 | 13.8 |  | 5.18 | | 0.000 |  | 1,257 | 1,214 | 3.7 |  | 1.12 | 0.264 |
| (Apgar score at 5 minutes) | M | 1,264 | 1,271 | -0.6 | 95.5 | -0.11 | | 0.909 |  | 1,242 | 1,259 | -1.5 | 58.5 | -0.32 | 0.749 |
|  |  |  |  |  |  |  | |  |  |  |  |  |  |  |  |
| birth_asphyxia | U | .0501 | .01748 | 18.1 |  | 7.55 | | 0.000 |  | .04957 | .05325 | -1.7 |  | -0.48 | 0.634 |
| (Birth asphyxia) | M | .0501 | .04697 | 1.7 | 90.4 | 0.32 | | 0.750 |  | .04679 | .04498 | 0.8 | 50.8 | 0.19 | 0.853 |
|  |  |  |  |  |  |  | |  |  |  |  |  |  |  |  |
| acidosis | U | .01357 | .00845 | 4.9 |  | 1.71 | | 0.087 |  | .01401 | .02367 | -7.1 |  | -1.88 | 0.061 |
| (Acidosis) | M | .01357 | .01461 | -1.0 | 79.6 | -0.19 | | 0.846 |  | .01415 | .00762 | 4.8 | 32.4 | 1.35 | 0.178 |
|  |  |  |  |  |  |  | |  |  |  |  |  |  |  |  |
| infection | U | .08246 | .02605 | 25.1 |  | 10.73 | | 0.000 |  | .08297 | .08059 | 0.9 |  | 0.25 | 0.800 |
| (Infection) | M | .08246 | .07829 | 1.9 | 92.6 | 0.34 | | 0.737 |  | .08052 | .07871 | 0.7 | 23.9 | 0.14 | 0.886 |
|  |  |  |  |  |  |  | |  |  |  |  |  |  |  |  |
| birth_injury | U | .01566 | .00401 | 11.8 |  | 5.55 | | 0.000 |  | .01616 | .00947 | 6.0 |  | 1.93 | 0.053 |
| (Birth injury) | M | .01566 | .01357 | 2.1 | 82.1 | 0.38 | | 0.704 |  | .01632 | .01922 | -2.6 | 56.7 | -0.47 | 0.638 |
|  |  |  |  |  |  |  | |  |  |  |  |  |  |  |  |
| syndrome_diabetes | U | .09708 | .01539 | 36.0 |  | 19.71 | | 0.000 |  | .09483 | .04521 | 19.5 |  | 6.59 | 0.000 |
| (Syndrome_of infants of mother with diabetes) | M | .09708 | .10717 | -4.4 | 87.6 | -0.73 | | 0.466 |  | .09249 | .1012 | -3.4 | 82.5 | -0.63 | 0.528 |
|  |  |  |  |  |  |  | |  |  |  |  |  |  |  |  |
| hypoglycaemia | U | .15658 | .0209 | 49.1 |  | 28.04 | | 0.000 |  | .15194 | .06308 | 29.0 |  | 10.00 | 0.000 |
| (Hypoglycaemia) | M | .15658 | .16597 | -3.4 | 93.1 | -0.56 | | 0.576 |  | .14472 | .14436 | 0.1 | 99.6 | 0.02 | 0.982 |
|  |  |  |  |  |  |  | |  |  |  |  |  |  |  |  |
| underfeeding | U | .03236 | .00446 | 20.9 |  | 12.34 | | 0.000 |  | .03341 | .01988 | 8.4 |  | 2.71 | 0.007 |
| (Underfeeding) | M | .03236 | .03166 | 0.5 | 97.5 | 0.09 | | 0.931 |  | .03373 | .02648 | 4.5 | 46.4 | 0.91 | 0.363 |
|  |  |  |  |  |  |  | |  |  |  |  |  |  |  |  |
| respiratory_disorder | U | .16493 | .03982 | 42.2 |  | 19.29 | | 0.000 |  | .16595 | .10497 | 17.9 |  | 5.62 | 0.000 |
| (Respiratory disorder) | M | .16493 | .15379 | 3.8 | 91.1 | 0.67 | | 0.506 |  | .16322 | .15778 | 1.6 | 91.1 | 0.32 | 0.751 |
|  |  |  |  |  |  |  | |  |  |  |  |  |  |  |  |
| cardiovascular_disorder | U | .01566 | .00247 | 13.9 |  | 7.87 | | 0.000 |  | .01616 | .01101 | 4.5 |  | 1.40 | 0.162 |
| (Cardiovascular_disorder) | M | .01566 | .01427 | 1.5 | 89.4 | 0.25 | | 0.802 |  | .01632 | .01523 | 0.9 | 78.9 | 0.19 | 0.852 |
|  |  |  |  |  |  |  | |  |  |  |  |  |  |  |  |
| seizure_intracranial_haemorrhage | U | .00626 | .00168 | 7.3 |  | 3.38 | | 0.001 |  | .00647 | .00959 | -3.5 |  | -0.94 | 0.347 |
| (Neonatal convulsion or intracranial haemorrhage) | M | .00626 | .00522 | 1.7 | 77.2 | 0.30 | | 0.763 |  | .00653 | .00435 | 2.4 | 30.3 | 0.63 | 0.526 |
|  |  |  |  |  |  |  | |  |  |  |  |  |  |  |  |
| preeclampsia | U | .11273 | .0401 | 27.6 |  | 11.24 | | 0.000 |  | .1153 | .06639 | 17.1 |  | 5.51 | 0.000 |
| (Preeclampsia) | M | .11273 | .11169 | 0.4 | 98.6 | 0.07 | | 0.942 |  | .11425 | .11607 | -0.6 | 96.3 | -0.12 | 0.903 |
|  |  |  |  |  |  |  | |  |  |  |  |  |  |  |  |
| diabetes | U | .12839 | .04163 | 31.5 |  | 13.17 | | 0.000 |  | .12608 | .08154 | 14.6 |  | 4.60 | 0.000 |
| (Diabetes) | M | .12839 | .13814 | -3.5 | 88.8 | -0.63 | | 0.531 |  | .12405 | .13275 | -2.9 | 80.5 | -0.56 | 0.577 |
|  |  |  |  |  |  |  | |  |  |  |  |  |  |  |  |
| haemorrhage_early | U | .04802 | .03739 | 5.3 |  | 1.72 | | 0.086 |  | .04741 | .04024 | 3.5 |  | 1.05 | 0.295 |
| (Haemorrhage in early pregnancy) | M | .04802 | .04558 | 1.2 | 77.1 | 0.25 | | 0.801 |  | .04788 | .04498 | 1.4 | 59.6 | 0.30 | 0.768 |
|  |  |  |  |  |  |  | |  |  |  |  |  |  |  |  |
| haemorrhage_ante_intrapartum | U | .03862 | .01838 | 12.2 |  | 4.59 | | 0.000 |  | .03879 | .02509 | 7.8 |  | 2.47 | 0.014 |
| (Antepartum/intrapartum haemorrhage) | M | .03862 | .02992 | 5.2 | 57.0 | 1.05 | | 0.296 |  | .03808 | .03119 | 3.9 | 49.7 | 0.81 | 0.419 |
|  |  |  |  |  |  |  | |  |  |  |  |  |  |  |  |
| infection_urinary_genital | U | .08142 | .05164 | 12.0 |  | 4.12 | | 0.000 |  | .07974 | .06012 | 7.7 |  | 2.35 | 0.019 |
| (Infection of genitourinary tract in pregnancy) | M | .08142 | .08212 | -0.3 | 97.7 | -0.06 | | 0.956 |  | .08052 | .08125 | -0.3 | 96.3 | -0.06 | 0.955 |
|  |  |  |  |  |  |  | |  |  |  |  |  |  |  |  |
| premature_rupture_membrane | U | .19833 | .09556 | 29.3 |  | 10.67 | | 0.000 |  | .20151 | .13728 | 17.2 |  | 5.30 | 0.000 |
| (Premature rupture of membrane) | M | .19833 | .19624 | 0.6 | 98.0 | 0.11 | | 0.909 |  | .20131 | .18462 | 4.5 | 74.0 | 0.91 | 0.365 |
|  |  |  |  |  |  |  | |  |  |  |  |  |  |  |  |
| infection_amniotic_sac | U | .01566 | .00913 | 5.9 |  | 2.10 | | 0.036 |  | .01509 | .01183 | 2.8 |  | 0.86 | 0.391 |
| (Infection of amniotic sac and membrine) | M | .01566 | .01566 | 0.0 | 100.0 | -0.00 | | 1.000 |  | .01415 | .01451 | -0.3 | 88.8 | -0.07 | 0.948 |
|  |  |  |  |  |  |  | |  |  |  |  |  |  |  |  |
| placenta_praevia_abruption | U | .01983 | .00783 | 10.3 |  | 4.14 | | 0.000 |  | .02047 | .01243 | 6.3 |  | 2.04 | 0.042 |
| (Placenta praevia or abruption placetae) | M | .01983 | .01705 | 2.4 | 76.8 | 0.45 | | 0.651 |  | .02067 | .01523 | 4.3 | 32.4 | 0.88 | 0.380 |
|  |  |  |  |  |  |  | |  |  |  |  |  |  |  |  |
| birth_calendar_year | U | 2007.3 | 2007 | 6.3 |  | 1.94 | | 0.053 |  | 2007.3 | 2007.6 | -8.5 |  | -2.46 | 0.014 |
| (Birth year) | M | 2007.3 | 2007.6 | -7.7 | -21.6 | -1.69 | | 0.091 |  | 2007.3 | 2007.2 | 2.7 | 68.6 | 0.58 | 0.559 |
|  |  |  |  |  |  |  | |  |  |  |  |  |  |  |  |
| income_family | U | 2,394 | 2,526 | -11.7 |  | -3.50 | | 0.000 |  | 2.403 | 2.508 | -9.3 |  | -2.63 | 0.008 |
| (Income of family) | M | 2,394 | 2,444 | -4.4 | 62.2 | -0.97 | | 0.331 |  | 2,406 | 2,438 | -2.8 | 69.6 | -0.61 | 0.543 |
|  |  |  |  |  |  |  | |  |  |  |  |  |  |  |  |
| maternal_age | U | 27.609 | 27.743 | -2.1 |  | -0.64 | | 0.525 |  | 27.63 | 27.638 | -0.1 |  | -0.03 | 0.973 |
| (Maternal age) | M | 27.609 | 27.77 | -2.5 | -20.3 | -0.53 | | 0.595 |  | 27.655 | 27.608 | 0.7 | -510.9 | 0.15 | 0.880 |
|  |  |  |  |  |  |  | |  |  |  |  |  |  |  |  |
| parity_maternal | U | .4405 | .51044 | -14.0 |  | -4.30 | | 0.000 |  | .44073 | .43231 | 1.7 |  | 0.49 | 0.623 |
| (Parity) | M | .4405 | .42658 | 2.8 | 80.1 | 0.61 | | 0.539 |  | .43961 | .44106 | -0.3 | 82.8 | -0.06 | 0.950 |
|  |  |  |  |  |  |  | |  |  |  |  |  |  |  |  |
| age_bilirubin_test | U |  |  |  |  |  | |  |  | 4,591 | 6,030 | -35.7 |  | -8.42 | 0.000 |
| (Age at time of bilirubin measurement) | M |  |  |  |  |  | |  |  | 4.605 | 4,469 | 3.4 | 90.5 | 1.12 | 0.264 |
|  |  |  |  |  |  |  | |  |  |  |  |  |  |  |  |
| quartile_bilirubin | U |  |  |  |  |  | |  |  | 3,392 | 2,416 | 97.6 |  | 26.13 | 0.000 |
| (Gestational age- and age-specific quartile of bilirubin) | M |  |  |  |  |  | |  |  | 3,386 | 3,406 | -1.9 | 98.0 | -0.48 | 0.630 |

^a:^ Mean was calculated using the variable values in parentheses (Sex: Girls (1), Boys (2); Gestational age (week): 35 (35), 36 (36), 37 (37), 38 (38), >=39 (39); Apgar score at 5 minutes: 09--10 (1), 7--8 (2), 0-6 (3), Missing (9); Birth year: 2002-2006 (2002), 2007-2011 (2007), 2012-2016 (2012); Family income (quartiles): first/lowest quartile (1), second quartile (2), third quartile (3), fourth quartile (4); Maternal age (years): <25 (12), 25-29 (25), 30-34 (30), 35-39 (35), >=40 (40); Age at time of bilirubin measurement: 1st day (1), 2nd day (2), 3rd day (3), 4th day (4), 5-7 days (5), 8-14 days (8), 15-21 days (15), 22-28 days (22); Gestational age- and age-specific quartile of bilirubin: same as family income; rest of variables: No (0), Yes (1)).

^b:^ The standardised % bias is the % difference of the sample means in children with phototherapy and children without phototherapy as a percentage of the square root of the average of the sample variances in the two groups

Children born alive at Aarhus University Hospital between 1.1.2002 and 30.11.2016 in the Danish Medical Birth Registry (n=71,781)

Exclusion of children

- multiple births (n=3,850),

- a missing (n=461) or unlikely (<200 g) birth weight (n=11),

- died in the neonatal period (n=201),

- emigrated from Denmark in the neonatal period (n=32)

- gestational age 22-34 (n=1816)

Study population (n=65,365)

Supplementary Figure 1. The framework of identifying the study population
